# Preferences for an eHealth tool to support physical activity and exercise training in COPD: a qualitative study from the viewpoint of prospective users

**DOI:** 10.1186/s12890-023-02353-3

**Published:** 2023-02-13

**Authors:** Pernilla Sönnerfors, Kirsti Skavberg Roaldsen, Sara Lundell, Annika Toots, Karin Wadell, Alexandra Halvarsson

**Affiliations:** 1 Division of Physiotherapy, Department of Neurobiology, Caring Sciences and Society, Karolinska Institutet, Huddinge, Sweden; 2 Women’s Health and Allied Health Professionals Theme, Medical Unit Occupational Therapy and Physical Therapy, Karolinska University Hospital, Stockholm, Sweden; 3 Department of Research, Sunnaas Rehabilitation Hospital, Nesoddtangen, Norway; 4 Faculty of Health Sciences, UiT The Arctic University of Norway, Tromsø, Norway; 5 Department of Community Medicine and Rehabilitation, Physiotherapy, Umeå University, Umeå, Sweden; 6 Department of Community Medicine and Rehabilitation, Occupational Therapy, Umeå University, Umeå, Sweden

**Keywords:** Telerehabilitation, Telemedicine, Internet use, Health literacy, Communication

## Abstract

**Background:**

Despite well-known positive effects of pulmonary rehabilitation, access is limited. New strategies to improve access are advocated, including the use of eHealth tools.

**Objectives:**

The aim of this study was to explore prospective users’ preferences for an eHealth tool to support the self-management of physical activity and exercise training in COPD.

**Methods:**

A qualitative research design was applied. Data was collected in six, audio recorded, digital co-creation workshops, which were guided by a participatory and appreciative action and reflection approach. A total of 17 prospective users took part in the process, including people with COPD (n = 10), relatives (n = 2), health care givers (n = 4) and a patient organization representative (n = 1). During the workshops, pre-selected relevant topics to exploring end-users’ preferences for eHealth support in self-management in COPD were discussed. The workshops were recorded and transcribed. Data was analysed using inductive qualitative content analysis.

**Results:**

The overarching theme “fusing with, rather than replacing existing support structures” was uncovered when the two-sided relationship between positive expectations towards digital solutions and the fear of losing access to established rehabilitation systems, emerged in the discussions. Three categories were identified, focused on wishes for an evidence-based support platform of information about COPD, a well-designed eHealth tool including functionalities to motivate in the self-management of physical activity and exercise training, and requirements of various forms of support. Co-creators believed that there were clear benefits in combining the best of digital and existing support systems.

**Conclusions:**

Co-creators viewed an eHealth tool including support for physical activity and exercise training as a valuable digital complement to the now existing rehabilitation services. A future eHealth tool needs to focus on user-friendliness and prospective users’s requests.

## Background

Chronic obstructive pulmonary disease (COPD) is a prevalent disease, estimated to be the third leading cause of death in the world [1, 2]. Common symptoms are shortness of breath (dyspnea), productive cough, fatigue, and decreased physical activity level [3, 4]. Several important prognostic factors have been established, where a person’s level of physical activity has been shown to be the strongest predictor of all-cause mortality in people with COPD [5]. Physical activity defined as “any bodily movement produced by skeletal muscles that results in energy expenditure beyond resting energy” [6] whereas the characteristics of physical exercise training are described as “any physical activity that is planned, structured, repetitive, and intended for achieving physical fitness” [7]. Physical activity and exercise training are considered to be the cornerstone of pulmonary rehabilitation [8].

Treatment guidelines recommend education, assessments, and individualized interventions to be included in pulmonary rehabilitation which should be offered to all people with COPD [9]. The positive impact of pulmonary rehabilitation on outcomes, such as health-related quality of life and physical capacity, is independent of disease severity [10–12].

Behavioral change is an important part of pulmonary rehabilitation and combining education with an individualized action plan has been shown to be beneficial [13]. To facilitate long-lasting behavioral changes, self-management strategies are recommended [13]. In addition, an interdisciplinary approach is often required to help people with COPD manage the disease and change their physical activity behavior [14]. Each member of the interdisciplinary COPD team (e.g., physiotherapists, physicians, nurses, occupational therapists, psychologists, social workers, nutritionists) has a unique and important role in the support [10].

Even though the positive effects of pulmonary rehabilitation are well-known, in Sweden it has been shown the access is limited to only a minority of the COPD population [15]. Even when exercise training is available the attendance is poor, due to individual and structural barriers, for example travel time and distance [16, 17]. This emphasizes the importance of finding strategies for improving access to pulmonary rehabilitation, including physical activity and exercise training. Electronic health (eHealth) tools can be used to support exercise training and physical activity in COPD, and improvements have been shown, both in physical activity level [18–21] and in physical capacity [20, 22–27]. The use of eHealth tools to support exercise training has also shown positive results regarding technology usability, safety, and the acceptance of the delivery mode and components [18, 19, 22, 25, 28–30]. However, low adherence to exercise training using eHealth has also been showed [31]. Health care providers have expressed that using eHealth tools could improve the continuity of pulmonary rehabilitation programs [23], an important aspect since long-term benefits decrease over time [32, 33]. The perceptions of using eHealth tools have been positive among people with COPD [34], more so than among health care providers [35]. However, the complexity of the implementation process has been described as high due to technological and/or organizational limitations [36]. In Sweden, where most people with COPD have a high degree of access to the Internet and to technical equipment, eHealth could be a suitable alternative since many use the Internet frequently and consider themselves likely to use an eHealth tool for COPD [37].

In order to make an intervention or product more suitable and appealing to prospective users and their settings, it is appropriate to use a process where the users are involved and participate in the development [38]. To further investigate the issue of how to make a suitable eHealth tool, for pulmonary rehabilitation for people with COPD, our research group conducted this co-creation study. The aim of this study was to explore prospective users’ preferences for an eHealth tool to support the self-management of physical activity and exercise training in COPD.

## Methods

### Study design

This study had a qualitative research design using workshops in a digital setting. The study was part of a larger project using co-creation to develop an eHealth tool for support in pulmonary rehabilitation [39]. The procedure adhered to the recommendations and principles for co-creation described by Leask et al. [38] and was guided by participatory and appreciative action and reflection (PAAR) [40]. The reporting of this study followed guidelines for reporting qualitative studies [41] and a checklist for studies with user involvement [42].

### Participants

A convenience sample of participants was recruited from hospitals, primary care and from researchers’ networks in two public health care regions in Sweden. The specific population, which the intervention is targeting (end-users), as well as other groups that will later be involved in the implementation and use of the developed product were invited to the co-creation process [38]. Participants were purposely selected to achieve variation in sex, age, disease severity and living situation (e.g., urban or rural area). Eligibility criteria for participation were (1) end-users with a COPD diagnosis according to the global initiative for chronic obstructive pulmonary disease (GOLD) [3], or end-users who are health care providers (e.g., physiotherapists, COPD-nurses, physicians) with experience in pulmonary rehabilitation within primary or specialty care, (2) being able to manage the functions of a smartphone, computer or tablet, and (3) living in the public health care regions of Stockholm or Västerbotten, Sweden. Furthermore, other participants invited were relatives (of both sexes) to persons with COPD, a patient representative from the National Heart and Lung Association in Sweden and a software developer from Umeå University. Researchers (SL, AT, PS) moderated the workshops.

### Data collection

Background information on the participants was collected by phone and e-mail. The background included information on (for people with COPD), years with COPD, lung function (FEV_1_; Forced Expiratory Volume in one second, FEV%; ratio of FEV_1_ to Forced Vital Capacity), COPD symptoms (COPD Assessment Test, CAT) [43], information on occupation and living conditions (e.g., occupational pension, disability pension, gainful employment), level of physical activity (“activity minutes” and intensity level) [44], gender, and public health care region (Stockholm or Västerbotten). Information on employment and years in the profession was collected from the patient representative from the National Heart and Lung Association in Sweden, and from the health care providers.

The six workshops were hosted digitally on a cloud platform for video communication and virtual conferences by Zoom Video Communications [35]. The workshops were held 2–4 weeks apart and each workshop lasted between 1.5 and 2 h. During the workshops, that were recorded, pre-selected topics relevant to exploring end-users’ preferences for eHealth support in self-management in COPD were discussed. Participants with COPD, relatives and moderators participated in all the workshops, while health care providers, and the patient organization representative were invited to participate in three of the workshops. The topics of the six workshops were: (1) introduction of methods and objectives and presentation of co-creators, (2) important components of pulmonary rehabilitation, (3) preferences regarding the design and content of the eHealth tool, (4) physical activity and exercise training in the eHealth tool, (5) support and motivation for behavior change, and (6) the COPD-team, prototype, and conclusion. The topics were addressed with pre-recorded films, digital lectures, and home assignments to up-skill participants. Each workshop was moderated and consisted of both whole group and small group discussions. In the first workshop the group discussed the PAAR method and decided together on the rules and responsibilities in the workshop sessions. Each workshop began with a summary and a short reflection from the previous workshop discussions and its content and accuracy were refined by the co-creators (e.g., when, and how to speak, and to respect each other’s opinions). A detailed description of the workshop process is provided by Lundell and Toots et al. [39].

### Data analysis

A qualitative content analysis with an inductive approach as described by Graneheim and Lundman [45] was used for the data analysis. The audio recordings from all the workshops (whole group and small groups) were transcribed by a professional transcriber. The transcripts were pseudonymized with letter codes (pseudonyms). In the process where the data was coded, a Microsoft Excel file was used.

Initially the transcripts were read through repeatedly to generate an overall impression of the data. Sections of text relevant to the aim of this study were extracted, and from these sections, texts were divided into meaning units (sentences and paragraphs). Reflective discussions within the research group were conducted around any confusing or unclear meaning unit and resolved together. The meaning units were condensed and labeled with a code. The first cycle of coding was descriptive in nature and the codes were sorted into common subcategories and categories. The second cycle of coding involved consolidating, renaming, and eliminating codes not relevant, always bearing the research aim in mind. The manifest analysis with the visible, obvious components was performed by PS in close cooperation with AH, KSR, and KW, and involved continuous back-and-forth movements from the whole text to parts thereof. In Table 1, examples of the coding strategy and the steps in the manifest analysis, including meaning units (sentences and paragraphs), condensed meaning units, codes, subcategories, and categories are presented. The latent analysis was performed by PS in close cooperation with all the authors in different constellations of groups. This part of the analysis also involved continuous back-and forth movements from parts of the texts and with interpretative discussions. To achieve trustworthiness in qualitative research, the important concepts of dependability, transferability, and credibility were considered. Dependability concerns any factors that could cause instability in the data and any variations introduced by the researcher during the data analysis. Visualizing the transferability of a study is possible when, for example, the context, the selection of participants and their characteristics, the data, and analysis are transparent and clearly described as in this present study. Credibility was considered both when participants were selected, during data collection at the workshops, and in the data analysis [45]. To ensure credibility in this present study, recurrent meetings were held within the whole group of authors regarding the most credible analysis and interpretation of the findings [45]. Codes, subcategories, main categories, and overarching theme were debriefed and discussed within the group of all authors until consensus was reached. The authors’ complementary competencies and perspectives were of great importance to the analysis. In this study, the data analysts were physiotherapists with specialist competence and clinical expertise in COPD and exercise training (PS, KW), scientific expertise in COPD (SL, KW), in eHealth (AH, SL, PS, KW), in exercise training/rehabilitation (AH, KSR, AT, KW), in qualitative research (AH, SL, KSR), and scientific competence in co-creation (SL, AT, KW). During the discussions, the researchers critically reflected upon their prior understanding. Both data collection and data analysis were performed in Swedish. The citations were translated by a native English-speaking external researcher physiotherapist and thereafter reviewed by all the authors.

**Table 1:** Examples of the steps in the manifest analysis in this study

Meaning unit	Condensed meaning unit	Code	Sub-category	Category
Yes, if I think of that film with the older gentleman, that had three different exercise sets. If you listened to what he said, you can find opportunities for progression in it. You can sit in the chair and stand five times, ten times, twelve times, twenty times. You can tighten the strap in different ways, according to the instruction, a different number of times but increase all the time, if you have reached the goal, if you manage twelve times as he says in the film then you can increase to fifteen or twenty. So, the answer is there, but you have to think a little yourself as well WS5 Co-creator with COPD	Some knowledge is required to understand how to increase your own exercise training level	Exercise training progression	Exercise training principles	A requirement for information on evidence-based practice
It [Regular contact with a physiotherapist] has really been the best. You feel that it is not so easy to cheat if they have a check on you. But I have been good, I have not cheated, but you could do it if you did not have someone who checked how you feel WS2 Co-creator with COPD	The fact that the physiotherapist has close contact gives a feeling that someone is checking up on you. This reduces the risk of cheating with the exercise training and increases the motivation to carry out the exercise training	Frequent follow-ups	Continuity in follow-ups and support	A desire for continous follow-ups and support from health care providers, peers, and significant others
Yes I would actually like to say this home training program. Can't it come digitally like this so you can see it while you do it, something. Not that you just get a piece of paper with everything printed on it WS5 Co-creator with COPD	Wishes for a digital home exercise training program, so you can watch when you complete the exercise training	Digital exercise training program	Technical properties for individual settings	A call for a well-designed eHealth tool with high demands on individual adaptation

*COPD* Chronic obstructive pulmonary disease, *WS* Workshop

## Results

### Participants’ characteristics

A total of 17 co-creators participated in the study (eleven women, six men). Characteristics of the ten co-creators with COPD are presented in Table 2. Two relatives of persons with COPD were included (one woman, one man), both pensioners. Four of the co-creators represented health care providers (three women, one man), one physician, two physiotherapists, one COPD-nurse, with 5 to 36 years of experience within the profession (mean, ± SD: 20 ± 14). One was representing a patient organization (woman) and was a pensioner. Nine of the 26 persons asked to participate declined. Six of them were persons with COPD or relatives, who declined due to: unwillingness to participate in a group setting (n = 1), unknown reason (n = 2) and a fear of not being able to manage the functions needed in the digital setting (n = 3). In addition, three health care providers declined due to expected extra workload.

**Table 2:** Characteristics of the co-creators with COPD

Characteristics	Persons with COPD (n = 10)
Age, years, mean ± SD (min–max)	71.1 ± 10.8 (51–87)
Sex, women/men, (n)	6/4
Occupation, pensioner/gainful employment, (n)	9/1
Time since COPD diagnosis, years, mean ± SD (min–max)	9.6 ± 8.2 (1–24)
FEV_1_% predicted, mean ± SD (min–max)	49 ± 24 (20–91)
Public health care region, Stockholm/Västerbotten, (n)	5/5

*COPD* Chronic obstructive pulmonary disease, *FEV*_*1*_ Forced expiratory volume in one second, *SD* Standard deviation

The mean attendance rate at the workshops was 89% among persons with COPD and 100% among the other co-creators. Most dropouts from the workshops occurred in the last two workshops (n = 5) and were mostly related to personal reasons such as family issues or health-related [39].

### Qualitative content analysis

The qualitative content analysis of co-creators’ preferences for eHealth support in the self-management of physical activity and exercise training in COPD, resulted in one overarching theme comprising three categories and ten subcategories, see Table 3.

**Table 3:** The results, showing the overarching theme, categories and subcategories

Subcategories	Categories	Overarching theme
Broad base of disease related information	A requirement for information on evidence-based practice	Fusing with, rather than replacing existing support structures
Exercise training principles		
Accessible health care providers		
User-friendly technology and optional features	A call for a well-designed eHealth tool along with high demands for individual adaptation	
Technical properties for structure and planning		
Automatically generated responses, reminders and rewards		
Complement to usual rehabilitation	A desire for continous follow-ups and support from health care providers, peers, and significant others	
An assigned health care provider		
Continuity in follow-ups and support		
Support from peers and significant others		

#### Fusing with, rather than replacing existing support structures

This overarching theme was identified based on the two-sided relationship between positive expectations towards digital solutions and the fear of losing access to established rehabilitation systems that emerged in the interviews. The eHealth tool was seen as important and advantageous for self-management of physical activity and exercise training in COPD. As participants were reluctant to compromise, they wanted to keep the best parts of the usual rehabilitation and saw clear benefits of combining the best of digital and existing systems. The overarching theme illustrates the expectations that digital and existing options put together would lead to a content delivery at a higher level and thereby surpass current treatment systems in COPD. The people living with COPD had requirements on the eHealth tool being not only technically advanced and user-friendly, but also adjustable to fit the COPD user’s individual needs, all to receive a better, more flexible, and strengthened version of support in their pulmonary rehabilitation.

#### A requirement for information on evidence-based practice

This category summarizes the perceptions regarding the need for education and information within the eHealth tool to support self-management of physical activity and exercise training. The eHealth tool should comprise a broad and robust platform of evidence-based information about COPD. In addition to the advantages of increased knowledge, this was also seen as a way to increase the use of self-management strategies via the eHealth tool.

##### Broad base of disease related information

The eHealth tool should provide access to accurate information about COPD, including recommended physical activities and exercise training according to clinical expertise, the latest updated guidelines, and research. It was expressed that the information should be accessible to people with COPD, their next of kin and health care providers, meaning the information should be reachable without using any login or password. Moreover, it was a common view that the presentation of the information needs to be adapted to a general population. The information needs to be objective with factual content but without being intimidating, despite the seriousness of the content. Information on the anatomy and physiology of the healthy body was seen as important, both to help with understanding the impact of COPD on the body and mind and the effects of physical activity and exercise training. It was also suggested that information ought to be incorporated about common symptoms, such as increased secretions, coughing, breathlessness, affected appetite, and how muscle function is affected, as well as for example osteoporosis and depression. Furthermore, information on usual medication and their common side effects was also seen as important. Another highlighted request was to have quick access to information on how to act in an acute situation. Participants wished for a sort of “panic button” to press where they could get quick information on how to act when the breathing is highly affected, and one experiences alarming and panicking breathlessness.

*“Yes, I think it is great that how to react in a panic situation [during breathing difficulties] is brought up. That [information] is the absolute best, I think”*

*- Co-creator with COPD, workshop 3*

##### Exercise training principles

Easily understandable information concerning the purpose, the benefits of, and how to perform exercise training in COPD was highlighted by the participants. Information on both exercise training principles in general, exercise training programs and specific personalized programs was requested. How to adjust to a persons’ current physical capacity, including specific information on how to modify the training to one’s current physical status (i.e., exercise training intensity), was expressed to be needed.

*“One thing that we said was important was to become aware of, or get an insight into, which exercises are good for what. That way you get even more motivated about what you are supposed to exercise”*

*- Co-creator with COPD, workshop 4*

Having knowledge on when to increase or decrease the exercise training intensity and differences regarding strength training, endurance training, and interval training were all seen as important issues to be able to independently manage and execute the exercise training efficiently. Furthermore, information on how to control the breathing and shortness of breath (i.e., breathing techniques) was also expressed by the participants to be included as it is an essential part of the instructions of how to conduct exercise training in COPD.

##### Accessible health care providers

Participants expressed it to be important to access information on the existing group of health care providers working with COPD, i.e., the COPD team. Knowledge on which parts of COPD rehabilitation the team members oversee, and people with COPD are entitled to, was suggested to be provided in the eHealth tool. Connecting with and learning from the health care providers who have knowledge of COPD was viewed as positive. This connection with the COPD team members could provide important information on (and give access to) their area of competence and interventions. An eHealth tool could also be a support during teaching sessions, used by health care providers who do not feel totally confident in every aspect of COPD rehabilitation.

*“But I think that everything, both the physiotherapist, all the parts [contact with the COPD team members] is really important, they were that for me/…/ the things that I have done and learned from everyone here, those working at Pulmonary rehabilitation. So, I can breathe now, and I have learned pursed-lip breathing so that I can even go for short walks.”*

*- Co-creator with COPD, workshop 2*

#### A call for a well-designed eHealth tool and high demands on individual adaptation

Preferences of how the functionalities of the eHealth tool must be designed in order to enable motivation to conduct physical activity and exercise training are summarized in this category.

##### User-friendly technology and optional features

High standards regarding usability, optional personalized features, design, and data security were required. Being able to easily communicate, with different relevant persons (e.g., health care givers, peers, one’s next of kin, a training partner, or a personal trainer) when using the eHealth tool (for example with short messages) and to easily navigate were seen as important. The participants expressed a potential risk of the eHealth tool not being used if the technical aspects were too advanced to handle, or if the exercise training required advanced (gym) equipment. Discussions regarding the design revealed participants’ requirements on the content being presented clearly with instructions, films, and pictures. Films were seen by the participants as facilitating and motivating since they could be used to clearly illustrate exercise training instructions and/or be followed (simultaneously) during the exercise training session but could also be used when illustrating inspiring examples of people living with COPD. Participants also expressed that the films should preferably include actors with whom they could identify. The settings on the users’ own profile page, for people with COPD, must be adjustable as well as the settings used by the health care providers. Some important concerns regarding challenges in data security (personal data) were raised. Both regarding how to transmit data collected via an eHealth tool and how to safely access the collected data without risking the disclosure of personal data to unauthorized persons (peers in a group or personnel). Therefore, the entrance to a personalized page in the eHealth tool must be both easy and safe and probably require a personal login. Another concern regarding the potential extra time needed when using the eHealth tool was expressed by health care providers.

“*But then you have some other problems with writing patient records and contact networks in relation to an application… We have both technical challenges and patient record challenges”*

*- Co-creator, health care provider, workshop 5*

##### Technical properties for structure and planning

Technical properties in the eHealth tool to help with structure and planning of exercise training and physical activity were demanded by participants. Following a personal schedule, including day, time, and personalized exercise training for oneself or together with others was seen as helpful. The schedule could be helpful in making the exercise training part of a routine, like having a set alarm.

*“It is good to schedule things so that you can have some structure to follow. And you can use an app for that.”*

*- Co-creator with COPD, workshop 5*

Another important function discussed was the possibility of logging one’s own statistics to visualize the current level of physical activity, exercise training, and personal goals. Daily notes would highlight changes and could help to provide realistic expectations on oneself. Participants also perceived the logging as a positive way of “motivating oneself” since the latest goals are visible and positively pushing a person to reach the next one. Moreover, participants also wanted to keep notes on current health status that could highlight (or exclude) any indications of a COPD exacerbation, or the need for an adjustment of the exercise training level. The participants pointed out the importance of the technical properties used for structuring being optional and not feeling too demanding.

##### Automatically generated responses, reminders, and rewards

The eHealth tool should give reinsurance to the users regarding the appropriate exercise training level by sending automatically generated responses on, for example, the level of the conducted exercise training. There should also be reminders on when to do the exercise training. A reminder on adjusting the training level was requested to show up as soon as a certain exercise training level is reached. The reminder should include needed instructions on how to make an adjustment, related to their accomplishments. Being able to maintain a correct exercise training level, as judged by a health care professional, was seen as important for keeping up motivation. Also, a wish was expressed for automatic encouragements and rewards (for example golden stars, happy smileys, extra encouragements) after certain accomplishments (a goal reached). Some expressed that a competitive part or a sort of gaming feature might trigger their motivation to continue with the exercise training. Non-activity should also generate an automatic response from the eHealth tool with a question on the reason for the absence from planned activity, and recommendations to contact healthcare if the absence is due to a COPD deterioration.

*“It could be an option that you get a small, like [text message]‘We see that you haven’t taken a certain number of steps, is there some reason for this?’”*

*- Co-creator, relative to a person with COPD, workshop 3*

#### A desire for continuous follow-ups and support

This category summarizes participants’ perspectives of the support needed in the management of COPD both in existing settings and when used in the complementary eHealth tool. Participants expressed a need for support provided in many ways and from different people and that the support could include people with various roles and with different areas of competence. For example, people with own experiences of COPD, relatives to a person with COPD, health care givers with knowledge of exercise training with COPD or other valuable competence in relation to the disease and common barriers. The eHealth tool, with the incorporated flexibility in the settings, was seen as a valuable complement to existing support and follow-ups, all to manage the COPD related challenges and recommended physical exercise training.

##### Complement to usual rehabilitation

The participants viewed the use of an eHealth tool as being a part of, or a complement to, usual rehabilitation at the clinic. The eHealth tool could facilitate health care contacts for persons with COPD unable to go to the clinic for various reasons. Obstacles, such as time-consuming travel, a long travel distance, a risk of infections, and difficulties in finding a training facility at all suitable for COPD training, were raised as examples that could be reduced with an eHealth tool.

*“And that was also the reason why I stopped going to my classes [COPD training]. Because it became so difficult, when it’s far away and you have to get dressed and undressed and take patient transport and you have to walk so far when you get there…..It’s taking the whole day you know, and then it becomes too much trouble”*

*- Co-creator with COPD, workshop 4*

Participants also expressed that the eHealth tool could function as a documentation support when reporting to other health care providers and as an inspirational tool. Furthermore, the eHealth tool could be a flexible complement, as interruptions due to transfers to other health care providers could be avoided. The eHealth tool was therefore seen as a promising way of reducing health care provider obstacles related to the healthcare systems, for continuity in COPD rehabilitation.

##### An assigned health care provider

Participants expressed the importance of having an assigned health care provider, competent in COPD, to contact via the eHealth tool; the professional affiliation was not always of importance. They wanted this health care provider to have the role of supporting with self-management strategies in different situations related to their disease. Participants highlighted it as very important that the contact with the assigned health care provider must be personal and trustful.

*“I have a hard time with self-discipline so I would really like one, a contact person who checks up on me ….. have you done that? ...and …you need to do this now…like that…I think that we all need to be seen in some way. And if it were possible with such a contact, of some kind, that would be beneficial.”*

*- Co-creator with COPD, workshop 3*

##### Continuity in follow-ups and support

Having close, frequent, and regular follow-ups via the eHealth tool with their assigned health care provider was seen as important and could include, for example, health related assessments as the CAT [43]. For the follow-ups involving exercise training, participants wanted to have a set time plan. Regarding feedback on the exercise training, the participants wanted this from a physiotherapist and the feedback should include individual adjustments of the exercise training intensity (higher level/lower level) and training results (number of repetitions or sets, dyspnoea/leg fatigue on the Borg scale). Participants discussed the need for support, and how this differs during different phases of the disease or due to individual variations. Getting support at the start of an exercise training period was expressed by participants as essential, since this also incorporates support in how to execute the exercises correctly.

*“You know, if I for example do 12 repetitions and I feel like it doesn’t work at all, I can’t even manage 10. Then maybe I would like some contact with a physiotherapist to ask about what’s happening and why I can’t manage, that way I could get an explanation of it.”*

*- Co-creator with COPD, workshop 4*

Furthermore, receiving positive feedback on the conducted exercise training was thought to give extra stimulus. This was seen as a positive pressure and a way to facilitate the continuity of the exercise training until the next follow-up session. In order to give accurate feedback, the participants stated that health care providers should have access to personal registrations in the eHealth tool, before the planned follow-ups. The access to a personal registration was seen as essential since changes in a person’s health status could require the health care providers to respond quickly, for adjustment of exercise training level or of the set goals.

##### Support from peers and significant others

Different kinds of support were expressed as an important part of the eHealth tool. The support in the eHealth tool could also come via other persons with COPD (peers), from one’s next of kin, a training partner, or a personal trainer (here called significant others). Getting support in the eHealth tool with the planning of an activity and with the actual conducting of an activity or exercise training together with significant others were examples that could be included. Furthermore, support was desired in finding ways when interacting with significant others to improve one’s own inner motivation regarding coping, both regarding the disease and the exercise training.

*..“most of all I think the films were fantastic…to see in a concrete way how people with COPD can exercise and behave in relation to physical activity and to be shown what you should do with examples.”*

*- Co-creator with COPD, workshop 3*

Being part of an exercise training group lounged in the eHealth tool for people with COPD or having a “training buddy” was seen as very positive and a way of improving training motivation. In addition, a digital chat room exclusively for people with COPD was seen as a place to meet peers where COPD related issues (e.g., physical activity and exercise training) could be discussed, and also a place to advertise for a training buddy. A group setting was perceived as positive in many ways. Some positive areas discussed were that the group setting could provide a feeling of security, having friends to spur each other on and "compete against" each other but also against oneself and reduce feelings of loneliness. Identifying with like-minded people with COPD and sharing experiences with people who understand, in a setting where no one has to stand out, was seen as being very supportive.

*“But it is…like I said there is more drive when you are several people, in some way…and have a program to follow. I think that makes a big difference”*

*- Co-creator, relative to a person with COPD, workshop 3*

## Discussion

### Principal findings

This study has explored prospective users’ preferences for an eHealth tool to support self-management of physical activity and exercise training in COPD.

A variety of perspectives were expressed which together lead to a theme where an ideal eHealth tool was described. This should meet a high-standard and be a comprehensive eHealth tool including the requested supportive components, which could substantially add to the already existing support in COPD without removing any existing and working supportive components. This theme symbolises the views and wishes for a future eHealth tool to add content and support for self-management, focusing on physical activity and exercise training. Participants suggested a combination that substantially adds to the support existing today, and therefore this new ideal eHealth tool was interpreted as a request similar to a fantasy not completely realistic to fulfil. In this present study, it was seen as important for the eHealth tool to have manageable and user-friendly technical solutions and not be too demanding technically. The design of eHealth interventions is often focused on supporting the relationship between patients and their health care providers as an integrated part, not a separated system and not to replace the personal interaction between them [46]. In previous studies there have been positive perceptions and experiences on the use of eHealth tools [30, 47], e.g., regarding both the convenience of the received care [31], the content of exercise training programs [24], and on aspects of usability and adherence [35]. On the other hand, for some participants, the technology used in eHealth interventions has been shown to be perceived as difficult [20, 48], which are important findings to take in consideration during the creation of a new eHealth tool for support in COPD. In an annual survey on the Internet habits of the Swedish population (2021), it has been shown that older persons (aged > 80) feel that the development of technology and technical devices is too advanced and sometimes leaves them separated from society [49].

The findings from this present study are consistant with results from a reseach review showing the need for support in different areas for people with COPD, for example understanding their disease, managing symptoms, getting practical support, and living a healthy life with the disease [50]. Furthermore, the research review also identified some other areas of support needed, not shown in this present study, for example anxiety and depression, thinking about the future, financial concerns, and result [50]. An interdisciplinary approach is often required to increase adherence to recommended physical interventions [14] as it can be challenging to motivate people with COPD to change a behavior [13]. In this present study, participants expressed a need to obtain evidence-based information from members of the COPD team with clinical expertise and knowledge of the latest guidelines, in order to handle their self-management strategies. Increased knowledge about exercise training, its execution and purpose were thought to provide a feeling of certainty when conducting the exercise training. Having a certain level of knowledge has also previously been shown to be inspirational and increase self-management in physical activities and exercise training [34]. The results in this present study are also in accordance with a study indicating that people with COPD are willing to take a more active role in self-management using eHealth [51]. Information on how to adjust exercise training and how to act in acute situations (with breathlessness) was seen by participants in the present study to facilitate self-management. Furthermore, similar to findings by Tistad, et al. [52], where participants expressed benefits of films/videos regarding understanding, communicating knowledge, and motivating proper performance, in this present study films/videos were seen as easy to understand, helpful, and motivating for instructions. In this study the importance of user-friendly and easy technical solutions that can be adjusted according to the users’ personal preferences was highlighted. It has previously been demonstrated that, due to a lack of knowledge on how to use an eHealth app and the technology being too complicated and demanding, people avoid using eHealth apps at all [49]. Potential difficulty regarding data security was a concern raised in this present study, and these results reflect those of a recent Swedish report showing that the online collection of personal data is a common concern [49]. Of Swedish Internet users, four out of ten are worried that someone can access and read their digital medical records [49]. This is important to address and to consider in order to get eHealth users to feel safe. We found the concerns regarding the use and handling of data generated by the eHealth tool being time consuming for health care providers to be in accordance with a previous study also showing concern regarding the lack of resources, being a barrier to managing the data generated by the eHealth tool [53]. In order to be sure that patients who are receiving eHealth interventions can handle information given in the eHealth tool regarding measurements and results, they need to have a certain level of understanding both regarding technology and how to interpret results from measurements [53]. A previous study by our research group has shown that people with COPD in Sweden have a high degree of access to the Internet and technical equipment [37], but their ability to handle measurements and results from an eHealth tool is important to consider, and not yet fully known. Use of automatically generated responses to the logged data from users, as suggested in the present study, could also be a way of keeping up the correct training intensities and reducing the possible misinterpretations. In line with the findings in the present study where participants expressed a wish for frequent follow-ups via the eHealth tool, a previous study also showed that repeated contact via eHealth tools should be with the same health care provider if possible [34]. This was preferable since they then have greater knowledge about the person’s medical history and therefore could better identify and handle changes [34]. Participants in this present study also viewed the close and regular follow-ups as motivating for adherence to the recommended exercise training. But worth noting was the expressed importance of having a physiotherapist assigned for the questions regarding exercise training, which has also been previously shown to be important in other diagnoses when using eHealth for exercise training [54]. Having regular and structured phone sessions with health mentors such as trained nurses has also been shown to improve self-management in COPD [55].

### Strengths and limitations

By using a qualitative methodology, this study sought to gain an in-depth understanding of the prospective users’ perspectives of the planned eHealth tool for COPD. In order to achieve trustworthiness, it is important to describe the research procedures and methods [45], which was done in this present study and in addition the consolidated criteria for reporting qualitative studies (COREQ) [41] and guidance for reporting involvement of patients and the public (GRIPP2) [42] were used for guidance in the report and could be seen as a strength.

During the data analysis, all researchers critically reflected upon their prior understanding and how it might affect their perceptions and interpretations, which is important to “let the text talk” and not input meaning that is not there [45]. The qualitative researchers’ preunderstanding of the subject could be both a limitation and a strength. In this present study, the moderators all being physiotherapists could have been a limitation and another occupation represented could have been a strength. But on the other hand, having physiotherapists with clinical experiences and scientific expertise in multiple areas was an asset in terms of having different valuable perspectives during planning and the actual implementation, in the discussions between the workshops, and when analyzing and interpreting data.

A strength was the use of a co-creation method, aiming to improve the effectiveness of, and adherence to, the intervention [38]. Both when developing an eHealth tool and in the implementation phase, the involvement of prospective users may enhance usability [56]. The co-creation method together with the PAAR research approach [40] was chosen since it requires the participants to use their appreciative intelligence and to focus on the best of what is currently experienced. Therefore, the positive experiences are accentuated, and the positive possibilities embedded in a given situation are recognized. The PAAR research approach was repeated together with guidance of the participants in how to discuss the main topic in relation to their own positive experiences. Consequently, their preferences and wishes for a future eHealth tool were not restricted by real life limitations, such as finances, technical devices, secure data transmission, the general data protection regulation (GDPR), etc. resulting in perspectives regarding an ideal tool. This could be seen as a limitation since only good examples may be expressed. However, as participants spoke freely in the discussions, they also expressed concern and obstacles considering the use of an eHealth tool. In the convenience sample in this present study, there was a variation (sex, age, disease severity, urban or rural living) to encompass a wide spectrum of perspectives [45]. The sample size in this present study was relatively small (n = 17), which could have limited the representation of health care providers and relatives, which should be considered regarding transferability of the results. On the other hand, there was representation from different health care providers’ professions and variation in years in the occupation and health care region. The setting with digital workshops was initially an adjustment to the current restrictions related to the COVID-19 pandemic. The digital setting incorporated a potential risk of not reaching the digitally naïve prospective participants, since it could have been a reason for declining. In this present study, participants had different levels of digital experience; three of the initially approached persons declined participation due to fear of not being able to manage the digital functions needed in the digital setting. But a strength with the new setting was the opportunity to include participants regardless of geography and without risk of infections. These advantages have previously been reported to be incorporated in a digital setting, in addition to the time efficiency including both travels to the clinic and waiting time [34]. The possibility of participating in a joint group despite geography can also increase the transferability of the findings since a broader variety of participants over the nation was included in this study [45].

Some of the participants had very little or no experience of using digital technology when entering this present study. So, considering this digital naiveté in some participants, the access to technical support was important, at least initially. Previous research has shown that it is important to have access to competent and efficient IT support [34], especially in terms of getting help to get started and feel safe while using the technology [23].

## Conclusion

The co-creators in this study viewed an eHealth tool as a valuable digital complement to the now existing health care resources. There were high demands on the content and technical and design features of the eHealth tool. The challenges raised in the study were mainly regarding safe documentation, transmission, and storage of the personal data and the health care providers’ limited resources. Participants acknowledge the use of an eHealth tool to facilitate ownership in managing the disease, but this requires evidence-based information and support. The findings in this study could contribute to the future development of novel eHealth tools being more focused on user-friendliness and being adapted to the actual needs expressed by the prospective users.

## Data Availability

The datasets generated during and/or analyzed during the current study are not publicly available to preserve the privacy and anonymity of the participants, but are available from the corresponding author upon reasonable request.

## Abbreviations

COPD: Chronic obstructive pulmonary disease
COREQ: Consolidated criteria for reporting qualitative studies
eHealth: Electronic health
FEV_1_: Forced expiratory volume in 1 second
FEV_1_%_pred_.: FEV_1_ in percent of predicted value
GDPR: The general data protection regulation
GOLD: The global initiative for chronic obstructive pulmonary disease
GRIPP: Guidance for reporting involvement of patients and the public
PAAR: Participatory and appreciative action and reflection
